# Chimpanzee quiet hoo variants differ according to context

**DOI:** 10.1098/rsos.172066

**Published:** 2018-05-23

**Authors:** Catherine Crockford, Thibaud Gruber, Klaus Zuberbühler

**Affiliations:** 1 Department of Primatology, Max Planck Institute for Evolutionary Anthropology, Leipzig, Germany; 2 Budongo Conservation Field Station, Masindi, Uganda; 3 Swiss Center for Affective Sciences, University of Geneva, Geneva, Switzerland; 4 Department of Zoology, University of Oxford, Oxford, UK; 5 School of Psychology and Neuroscience, University of St Andrews, St Andrews, Fife, UK; 6 Cognitive Science Centre, University of Neuchâtel, Neuchâtel, Switzerland

**Keywords:** animal communication, chimpanzee, call diversification, cooperation

## Abstract

In comparative studies of evolution of communication, the function and use of animal quiet calls have typically been understudied, despite that these signals are presumably under selection like other vocalizations, such as alarm calls. Here, we examine vocalization diversification of chimpanzee quiet ‘hoos’ produced in three contexts—travel, rest and alert—and potential pressures promoting diversification. Previous playback and observational studies have suggested that the overarching function of chimpanzee hoos is to stay in contact with others, particularly bond partners. We conducted an acoustic analysis of hoos using audio recordings from wild chimpanzees (*Pan troglodytes schweinfurthii*) of Budongo Forest, Uganda. We identified three acoustically distinguishable, context-specific hoo variants. Each call variant requires specific responses from receivers to avoid breaking up the social unit. We propose that callers may achieve coordination by using acoustically distinguishable calls, advertising their own behavioural intentions. We conclude that natural selection has acted towards acoustically diversifying an inconspicuous, quiet vocalization, the chimpanzee hoo. This evolutionary process may have been favoured by the fact that signallers and recipients share the same goal, to maintain social cohesion, particularly among those who regularly cooperate, suggesting that call diversification has been favoured by the demands of cooperative activities.

## 1. Introduction

Within the framework of the evolution of communication, how and why some species have greater call diversity than others—and more context specificity of vocalizations—remain much discussed. One extensive debate has centred on the variety of context-specific vocalizations within a species' repertoire, such as how precisely primate calls refer to objects and events external to themselves [1–7]. Context specificity has been particularly well documented in alarm calls, for example the predator-specific vocalizations of vervet monkeys ([8,9] but see [5]). Various evolutionary scenarios have been proposed to explain the origins of this type of signalling behaviour [9–11]. For instance, kin selection predicts that callers will gain a fitness benefit, provided a costly behaviour, like producing conspicuous vocal behaviour in the presence of a predator, favours close genetic relatives [12]. However, alarm calls can also be directly beneficial for the caller, for example if calling fosters group-level defence or if it has aversive effects on the predator [9]. Alarm calls have also been well studied when examining the evolution of vocal diversity. One relevant line of research has demonstrated that if a prey species regularly encounters various predators that differ in their hunting behaviour, then this can lead to the evolution of acoustically distinct, predator-specific alarm calls, a process well documented in social carnivores [11,13] and non-human primates [9]. Predation pressure, in other words, can be one of the main causes for the evolution of call diversity.

However, the evolution of call diversity also seems to be favoured by social factors. Species living in complex social units are permanently caught between cooperation and competition, and communication plays a key role in navigating between these two forces. Illustrating this, chacma baboons produce grunts during a range of social interactions. Dominant females, for example, grunt during approaches to handle a subordinate female's infant [14,15]. When grunts are emitted, grunters are more likely to be permitted access to infants, suggesting that grunts aid predictability of benign intent [14,15]. This and other observations have led to the hypothesis that social complexity is one of the key drivers for the evolution of vocal behaviour [16,17]. The main argument is that if individuals are regularly challenged by competing with others over resources, then selection is likely to favour the evolution of signals to minimize costs.

Selection for call diversity may also emerge when coordination with other group members becomes essential [11], provided such social interactions confer fitness gains [18–22]. Furrer & Manser [23] suggest that species that require coordinated escape responses from predators may evolve more context-specific alarm calls than species that do not. Species that coordinate hunting are also expected to evolve specific signals to increase hunting success: chimpanzees produce acoustically distinct hunt barks only when they hunt monkeys [24], which observations suggest function to recruit group members [24,25]. A last example regards mutual grooming, a highly coordinated and cooperative activity that, in some species, can involve exposure to vulnerable body parts. Chimpanzees use a specific signal, lip smacking, which appears to facilitate this type of cooperative interaction [26].

A particularly common coordination problem in social animals is group travel, and many primates living in visually dense habitats have evolved specific vocal signals, contact calls, to help maintain proximity [27]. Positive selection may particularly shape contact call evolution in species when reproductive benefits are accrued through coalition formation, and vocalizations enhance cohesion between coalition partners, such as in chimpanzees. Contact calls can be loud, reaching across hundreds of metres, or quiet, reaching only 50–150 m. Examples of quiet calls used to maintain contact within social groups are the coo calls of Japanese macaques [28], the peeps of bonobos [29] or the ‘move’ grunts of chacma baboons [30] and vervet monkeys [31].

Chimpanzees (*Pan troglodytes*) are an interesting species to investigate coordination problems, given that they gain benefits from coalition formation when engaged in both within and between group competition [20,32,33]. Chimpanzees also live in low visibility forest such that vocalizations become key in predicting the behaviour of others. Additionally, they have a fission–fusion social system, which makes group travel a more difficult problem due to the continuously changing social units, which are mediated by differentiated association preferences [34–36]. Thus, chimpanzee travel units can range from large groups to small parties to solitary travel, which requires negotiation with adequate signals and flexibility [37,38]. To this end, long-distance calls (pant hoots) are likely to play an important role in promoting fusion and coordination, especially during feeding and travelling [37,39]. More recently, quiet ‘hoo’ calls have also been noted to aid coordination in relation to travel [40].

Chimpanzees emit hoos in at least three distinguishable contexts, when initiating or during travel (travel hoo), when stationary, particularly when resting or feeding (rest hoo) or when seeing hidden threats, such as snakes (alert hoo). Chimpanzees are more likely to emit travel and alert hoos when cooperation partners are present than absent [40,41]. Field experiments show that different responses are elicited from receivers after hearing either a rest or an alert hoo broadcast from a hidden speaker. Specifically, receivers engage in more search behaviour after hearing alert than rest hoos [42] and appear to take the hoo variant as an indicator of the caller's awareness of a threat [43]. In the current study, we focus on the acoustic features of chimpanzee hoo calls that can serve as potential carriers of contextual information.

## 2. Material and methods

### 2.1. Study site and subjects

Subjects were wild-living, habituated chimpanzees of the Sonso community in Budongo Forest, Uganda [44]. Vocalizations were recorded between February 2008 and September 2010 by two observers, T.G. and C.C., from adult and subadult chimpanzees of both sexes. T.G. mainly recorded calls in rest and travel contexts, whereas C.C. mainly recorded calls in alert and rest contexts. Out of a total of 77 chimpanzees, we obtained good quality hoo recordings from 29 individuals: 14 males (nine adults greater than 14 years, five subadults 10–14 years) and 15 females (11 adults greater than 13 years and four subadults 10–13 years) (table 1). Data are available in the electronic supplementary material, data file.

**Table 1.** Distribution of calls across subjects.

subject	sex	age	alert	rest	travel
BB	M	A		2	
FD	M	A		5	5
FK	M	S		4	5
HT	F	A	1	1	
HW	M	A		7	5
JN	F	A		6	4
KA	F	S	2		
KL^a^	F	A	3	4	5
KT^a^	M	A	5	9	5
KU	F	A		6	5
KW^a^	F	A	2	8	5
KY	F	A	2	2	
KZ^a^	M	S	2	12	5
MK	F	A		2	
ML	F	A		5	5
MS^a^	M	A	1	7	5
NB^a^	F	A	1	7	5
NK^a^	M	A	2	11	5
NR	F	S	1	1	
OK	F	A	3		
PS^a^	M	S	2	6	5
RE^a^	F	S	2	4	5
SQ^a^	M	A	4	7	5
TK	M	A	2	1	
VR	F	S	2		
ZF	M	A		7	5
ZG	M	S	1	2	
ZL^a^	M	S	2	6	5
ZM	F	A		5	5

^a^Subjects with calls in each context used to make the discriminant functions. Remaining individuals' calls were permuted into the analyses. N = 271 calls; 29 chimpanzees.

### 2.2. Recording

We recorded hoos opportunistically using either a Sennheiser directional microphone MKH416 or MKH418 microphone with a Marantz PMD-660 solid-state recorder, an external Sennheiser directional MKE-400 microphone attached to a Panasonic NV-GS 330 DV camera or a Panasonic NV-GS 330 DV camera with an internal microphone. In all cases, calls were digitized at a 44.1 kHz sampling rate and 16-bit sampling depth. Each time hoos were recorded, the signaller, date and context of calling were noted. Although, when listening to recordings, we could detect no obvious acoustic differences from hoos recorded on different devices, given that recording devices may cause slight acoustic variation, we controlled for the recording device used in statistical models (see below).

### 2.3. Behavioural context

We classified hoos according to their context of production. We classified calls as ‘alert hoos’, if emitted in response to a hidden threat, such as a sedentary viper, viper model or a wire snare. We classified calls as ‘rest hoos’ if emitted when resting. By contrast, we classified hoos as ‘travel hoos’ if emitted immediately before (on average 0.8 s prior departure; *N* = 15, range: 0–3.0 s) or during travel (*N* = 72). *N* = 7 cases where individuals failed to recruit other individuals for travel while producing hoos were also included [40]. (Audio recordings are included in the electronic supplementary material.)

### 2.4. Acoustic analyses

Any call of sufficient quality and produced in the three contexts was subjected to acoustic analysis. Selection criteria were that at least the lowest frequency band had to be clearly visible throughout the call with no overlap from the calls of other individuals, that the signaller could be clearly identified, that the context of production was unambiguous and that all acoustic variables (table 2) could be measured. A maximum of two calls per bout were measured, although these were never sequential neighbours. Calls from digital audio files or digital videos were analysed using the PRAAT software [45]. Sound files were lifted from digital video using a VLC player (VideoLan Project 2001).

**Table 2.** Acoustic differences of hoos across contexts: discriminant function scores for analyses 1 and 2. *F*_0_: fundamental frequency; analysis 1: contexts rest, travel and alarm; analysis 2: contexts: travel and alarm, enabling bout information to be included (inter-call interval). Italics: greater than 1 or less than −1 (highly influential).

acoustic variable	analysis 1 (three contexts)		analysis 2 (two contexts)
	discriminant function 1	discriminant function 2	discriminant function 1
call duration (log)	−*1*.*41*	−0.40	−*0*.*91*
maximum *F*_0_ (log)	−0.15	*4*.*22*	−*2*.*17*
drop in *F*_0_ (sqrt)	−0.17	−0.05	0.00
peak frequency position^a^ (sqrt)	0.08	−0.01	0.03
maximum *F*_0_ position^a^ (log)	0.01	0.13	−0.05
inter-call interval (sqrt)	—	—	−*3*.*81*

^a^As a proportion of call duration.

Hoos are relatively quiet calls, thus even when recording using a directional microphone in a range of 5–20 m from the signaller, the signal-to-noise ratio is too low to allow for extracting reliable measures using automated software programs. Thus, 10 acoustic variables describing temporal and frequency call parameters that could be reliably measured by hand were measured manually using PRAAT software with spectrograms made using a fast Fourier transform length of 256 points with Gaussian window, time step of 1000 and window length of 0.05 s.

We measured eight acoustic variables to characterize prominent temporal and frequency features of primate vocalizations (call duration, fundamental frequency (*F*_0_) at the start and end of the call as well as the maximum *F*_0_; peak frequency—the frequency (Hz) with the maximum amplitude; time to maximum *F*_0_ and peak frequency from the start of the call and inter-call interval). We derived an additional four acoustic variables to further characterize fundamental and peak frequency change through each call (*F*_0 drop_: *F*_0 max_ − *F*_0 end_; steepness of *F*_0 drop_: *F*_0 drop_/duration from *F*_0 max _− *F*_0 end_; position of the maximum *F*_0_: time to maximum *F*_0_/call duration; position of the peak frequency: time to peak frequency/call duration). For duration measures, we used the standard cursor function along the fundamental frequency, which was clearly visible in all recordings. We calculated *F*_0_ measures using the ‘Pitch Listing’ function, which measures the *F*_0_ at less than 0.01 s intervals. We used the spectral slice function in PRAAT as a double check for accuracy of the ‘Pitch Listing’ function. Maximum *F*_0_ was defined as the highest fundamental frequency in the call. Start and end *F*_0_ measures were taken within the first or last 0.05 s of the visible *F*_0_ band for the call, respectively. Changes in *F*_0_ across each call were measured by two variables: *F*_0_ drop was the drop in *F*_0_ from the maximum *F*_0_ to the end *F*_0_. Slope steepness was the rate of decrease in *F*_0_ from the maximum to the end *F*_0_. We measured two variables that captured the time point in the call in which maximum *F*_0_ and peak frequency occurred. Here, each time point was calculated as a proportion of call length. To measure time to peak frequency, the ‘Intensity Listing’ function was used together with the cursor function to first determine the position of the peak frequency within the call. Inter-call interval was the duration between two calls within the same unbroken sequence of calls of the same call type, measured from the end of the last call to the beginning of the next call.

### 2.5. Statistical analyses

Where required for assumptions of statistical tests, appropriate variable transformations were conducted to obtain symmetrical distributions prior to the analysis [46]. We log-transformed call duration, maximum *F*_0_ and position of maximum *F*_0_. We square root-transformed frequency drop, position of peak frequency and inter-call interval. The position of maximum *F*_0_, position of peak frequency, *F*_0_ start and *F*_0_ end were used to construct our key variables and remained untransformed. High correlations were found between max *F*_0_, start and end *F*_0_ as well as between *F*_0_ drop and slope steepness (Pearson's correlation: *r* > 0.7). We thus discarded slope steepness, start and end *F*_0_, and kept the remaining six variables in the analysis with variance inflation factor less than 2, showing acceptably small correlational propensity.

#### 2.5.1. Context effects on acoustic structure

To determine whether hoos emitted in the three different contexts could be acoustically differentiated, we conducted a permuted discriminant function analysis, permuting contexts within subjects (‘pDFA’, [46]). This procedure has been recommended to account for non-independence of calls due to repeated recordings from the same subjects. Calls with sufficient quality to measure target acoustic variables totalled *N* = 271 calls from 29 chimpanzees (table 1).

To derive the discriminant functions, we only included hoos from individuals that contributed to each context (pDFA1: *N* = 11 callers, pDFA2: *N* = 11 callers), using one randomly selected call per individual per context. Thus, 33 calls were used to derive the discriminant functions: *N* = 3 adult females, *N* = 5 adult males and *N* = 2 subadult males (table 1). All the remaining calls were then cross-classified using the derived discriminant functions. We ran two pDFAs: the first included only acoustic parameters that described single calls and the second also included a parameter that described call bout information (inter-call interval). Given that hoos in rest contexts are almost always emitted as single calls, rest hoos were omitted from the second pDFA. Calls used for cross-classification, in pDFA1, were 129 calls from the same individuals used to make the discriminant functions and 109 calls from 17 additional individuals. In pDFA2, for cross-validation, there were 23 additional calls from the same individuals used to make the discriminant functions and 55 calls from 14 additional individuals. To avoid that the result would unduly depend on a particular random selection, we created 100 such random selections and averaged the result. We included the selected acoustic parameters (five for DFA1 and six for DFA2, including inter-call interval) that described the temporal and frequency distribution characteristics of each call and had a variance inflation factor of less than 2.

We based our assessment of the discriminability of the three contexts on the percentage correctly cross-classified calls and used 10 000 permutations to estimate the *p*-value for discriminability. The pDFAs were conducted in R v. 3.2.5 [47] using a function (provided by R. Mundry), which is based on the function lda of the R package MASS [48].

#### 2.5.2. Controlling for age, sex, observer and recording methods on acoustic structure

To determine the influence of the context of call production in relation to other possible influencing factors on the acoustic structure of hoos, we conducted linear mixed models (LMMs; [49]). We conducted one model for each acoustic variable shown to be influential on the distribution of hoos across contexts in the pDFA (those with a discriminant function score greater than 1.0 or less than −1.0) (table 2). We used LMMs with a Gaussian error structure and identity link using R v. 3.2.5 [47] and the function glmer of the package lme4 [50]. Here, we used all hoos from each context (*N* = 271 calls and *N* = 29 subjects, table 1).

Each model tested the same set of fixed and random effects on each acoustic variable. Call context was our main variable of interest and was considered to be the test predictor. We added age (in years) and sex (male/female) as control predictors, given that we included calls from both males and females, adults and subadults in the model. We also added observer and recording device as control predictors, given that these varied across contexts. Two observers (C.C. or T.G.) recorded calls using different recording devices (Sennheiser directional microphone, video recorder internal microphone or external Sennheiser microphone attached to the video recorder). Since for two contexts we only had recordings of one observer, we were unsure whether the analysis would suffer from confounding effects or if we would be able to disentangle the respective influence of observer and context on call features. To address this question, we conducted simulations, which revealed that the LMM model used is indeed able to tease apart the effects of observer and context, revealing unbiased estimates (electronic supplementary material, figure S2).

Because subjects usually contributed calls to more than one category, subject identity was included as a random effect. No random slopes could be fitted, as all combinations of fixed and random effects had at least one instance when fewer than two different values of the fixed effects occurred per level of the random effects [51,52]. We tested the significance of the individual fixed effects by comparing the full model (comprising all fixed and random effects) with a respective reduced model (not comprising the test predictor) using likelihood ratio tests [53]. Model estimates were only considered if the full-null model comparison was significant. Model stability was assessed for all models by excluding the random effects one at a time and comparing the estimates for these data with those for the full dataset. This showed no influential subjects or events. Finally, given that we needed to run two models, one for each key acoustic variable, the LMMs conducted constituted multiple tests of the same calls. Thus, *p*-values were subjected to Bonferroni corrections, and significance was considered to be reached with *p* = 0.017.

Finally, to further examine the influence of call context on call bout information, particularly inter-call interval, we ran an LMM that included the two contexts that showed variation in inter-call interval: travel and alert contexts. Given that only single hoos are emitted in rest contexts, rest hoos had no inter-call interval and thus were excluded from this analysis. Here, we additionally included control predictors, sex and age. Owing to reduced power and complete separation issues, we did not include observer or recording device in this model.

## 3. Results

### 3.1. Context specificity of hoo types

**Figure 1.**
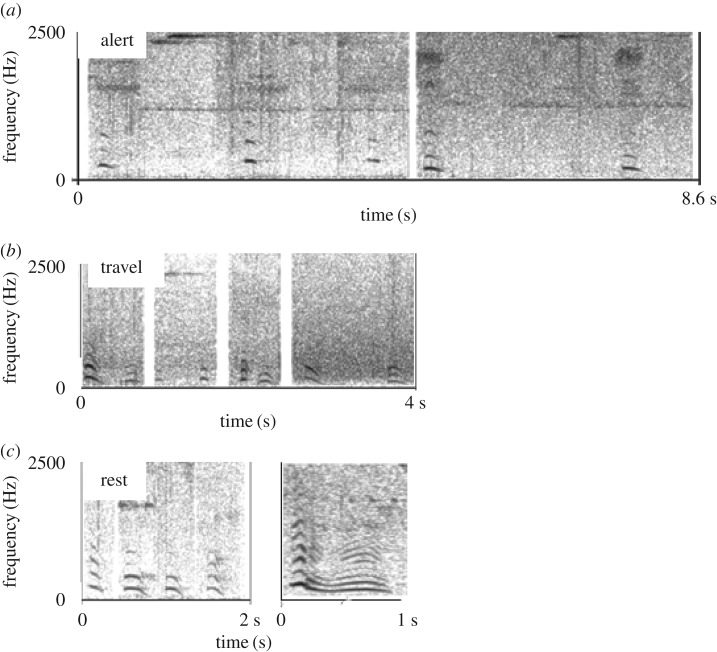
Spectrograms of the three hoo variants, including hoo sequences. (*a*) Alert hoos: two hoo sequences emitted by an adult male and female, respectively. (*b*) Travel hoos: four hoo sequences, emitted by two adult males and two adult females, respectively. (*c*) Five rest hoos, emitted by two adult males, two adult females and finally one adult male, respectively. Time and frequency scales are equivalent across spectrograms. Audio recordings are included in the electronic supplementary material.

**Figure 2.**
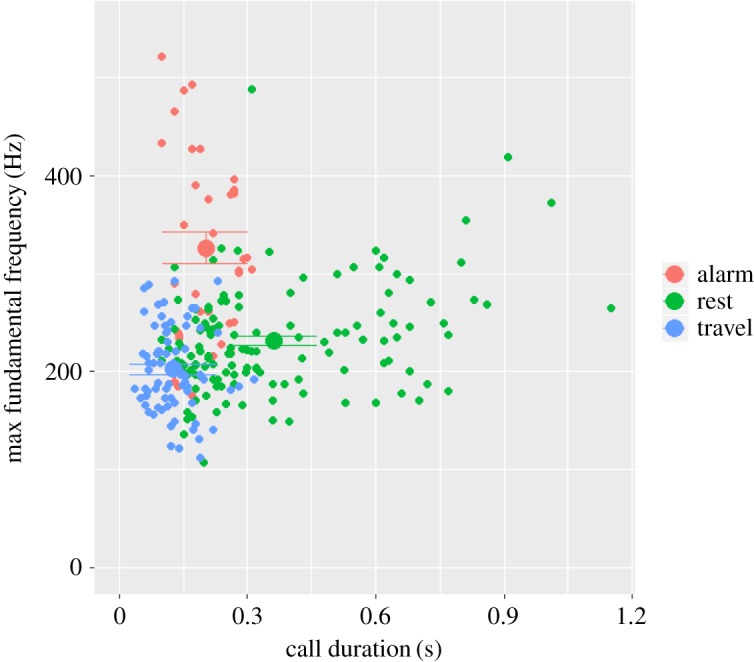
Classification of three hoo variants emitted in different behavioural contexts, delineated by two acoustic variables highly influential in permuted discriminant function analysis: call duration and maximum fundamental frequency (table 2). Group centroids with 95% confidence interval are shown.

Examining single calls from all three contexts together (alert, rest and travel, see figure 1 for spectrograms) showed reasonable discrimination of the hoo types based on five uncorrelated temporal and frequency parameters (pDFA: correct classification for cross-classified calls = 70.6%; expected correct classified calls = 35.8%; *p* = 0.001; figure 2 and table 2). Results from a single cross-validated DFA showed correct classification scores per context as follows: resting = 84.7%, travel = 83.0% and alert = 40.0%. Discriminant function loadings (table 2) and cross-classification scores showed that rest hoos discriminated well from travel hoos, having longer call duration. Alert hoos could be partially discriminated from the other two contexts having a higher maximum fundamental frequency. However, a subset of alert hoos did not discriminate well (figure 2).

**Figure 3.**
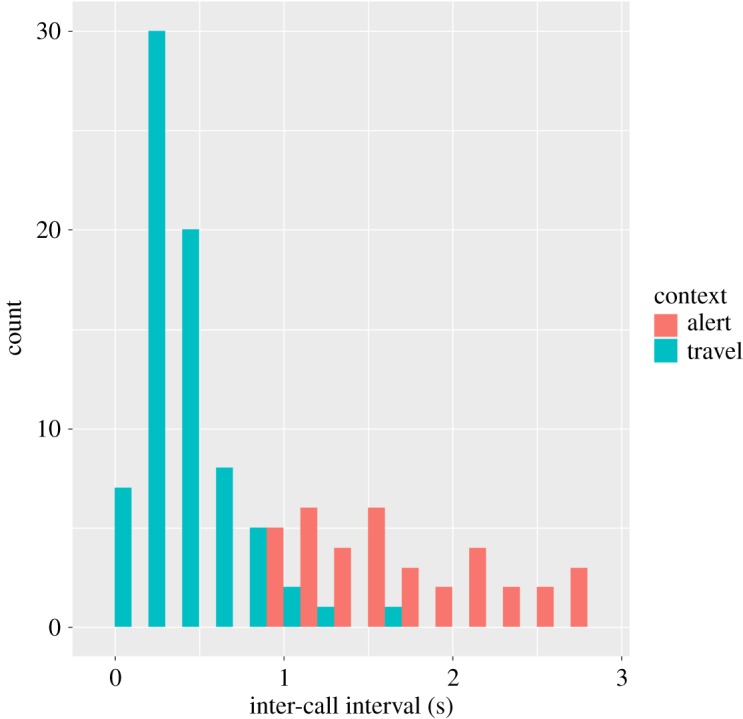
Influence of context on call interval in travel and alert contexts.

We conducted a second discriminant function analysis that included call bout information, specifically the duration between calls. Since rest hoos are almost always produced singly (134/137 cases, 97.8%) while travel (79/94 cases, 84.0%) and alert hoos (38/40 cases, 95.0%) are almost always produced in bouts of more than one call, we included only travel and alert hoos in the second analysis, in cases when more than one call was emitted. Thus, we included *N* = 112 calls in which 22 were selected to create the discriminant functions (from the same 11 chimpanzees as for analysis 1, table 1; *N* = 5 calls were omitted where bout information could not be reliably measured). Calls from two different contexts (alert and travel) could be discriminated well with the additional acoustic variable encoding call bout information (pDFA: correct classification for permuted cross-classified calls = 95.34; expected correct classified calls = 54.6%; *p* = 0.001; figure 3 and table 2). Correct classification scores per context were 97.4% for alert hoos and 97.3% for travel hoos. Discriminant function loadings (table 2) and cross-classification scores showed that alert hoos had longer inter-call intervals that travel hoos.

### 3.2. Acoustic differences in hoo types

We subjected the three acoustic variables (*F*_0_ max, call duration and inter-call interval) that were highly influential in the pDFA to further testing. Specifically, we conducted an LMM for each to determine the relative influence of test predictor, call context and control predictors on each acoustic variable. For each model, the full model was significant with respect to the null model (LMM: model significance against null model: *F*_0_ max as a response variable: *χ*^2^ = 45.07, d.f. = 2, *p* < 0.0001, three contexts, *N* = 271 calls from 29 subjects; call duration as a response variable: *χ*^2^ = 144.75, d.f. = 2, *p* < 0.0001, three contexts, *N* = 271 calls from 29 subjects; inter-call interval as a response variable: *χ*^2^ = 138.03, d.f. = 2, *p* < 0.0001; two contexts, *N* = 112 calls; 29 subjects). In both models, the acoustic variable tested was significantly influenced by the context of production of the call (table 3). We found no significant age or sex effects in either model. The observer had a significant influence on both *F*_0_ max (*χ*^2^ = 9.74, d.f. = 1, *p* = 0.002) and call duration (*χ*^2^ = 6.45, d.f. = 1, *p* = 0.011), while recording device effects were found for *F*_0_ max only (*χ*^2^ = 17.91, d.f. = 2, *p* = 0.0001; electronic supplementary material, figure S1). Here, it should be noted that the LMM separates variation attributed to the different predictors such that the influence of observer and device on the hoo acoustics cannot also account for context effects. In addition, our simulations showed that the influence of each predictor remained independent (electronic supplementary material, figure S2). The third model showed that the interval between calls is longer in alert than travel hoos (table 3 and figure 3).

**Table 3.** The influence of behavioural context on hoo acoustic properties: LMM full model results. Analysis 1 includes hoos from rest, travel and alert contexts: *N* = 271 calls from 29 chimpanzees. Analysis 2 includes hoos from travel and alert contexts: *N* = 112 calls from 29 chimpanzees. LMM full versus null model results: maximum *F*_0_ model: *χ*^2^ = 45.01, d.f. = 2, *p* < 0.000. Call duration model: *χ*^2^ = 144.75, d.f. = 2, *p* < 0.000. Inter-call interval model: *χ*^2^ = 138.03, d.f. = 1, *p* < 0.000. s: directional microphone + audio recorder; cs: video recorder + external microphone; c: video recorder; tg: observer T.G. After Bonferroni correction, *α* level is set to *p* = 0.017. Bold: *p* < 0.017.

acoustic variable	predictor variable	d.f.	*χ*^2^	*p*	*ß*	s.e.	*t*
analysis 1 (three contexts)							
maximum *F*_0_ (log)	intercept				5.74	0.09	
	call context	**2**	**45**.**10**	**<0**.**000**			
	—rest hoo				−**0**.**31**	**0**.**05**	−**5**.**88**
	—travel hoo				−**0**.**42**	**0**.**05**	−**7**.**00**
	—alert hoo				**0**	**0**	**0**
	sex (male)	1	0.71	0.40	0.00	0.04	0.85
	age	1	0.21	0.65	0.00	0.00	−0.46
	observer (tg)	**1**	**9**.**48**	**0**.**002**	−**0**.**13**	**0**.**04**	−**3**.**12**
	recording device (cs)	**2**	**17**.**91**	**0**.**0001**	−**0**.**15**	**0**.**09**	−**1**.**67**
	recording device (s)	**2**			**0**.**12**	**0**.**08**	**1**.**39**
call duration (log)	intercept				−1.62	0.18	
	call context	**2**	**144**.**75**	**<0**.**000**			
	—rest hoo				**0**.**63**	**0**.**12**	**5**.**24**
	—travel hoo				−**0**.**28**	**0**.**14**	−**2**.**00**
	—alert hoo				**0**	**0**	**0**
	sex (male)	1	3.01	0.08	0.12	0.07	1.76
	age	1	0.00	0.96	0.00	0.00	0.04
	observer (tg)	**1**	**6**.**35**	**0**.**01**	−**0**.**24**	**0**.**10**	−**2**.**53**
	recording device (cs)	2	0.48	0.80	−0.12	0.19	−0.62
	recording device (s)	2			−0.10	0.17	−0.62
analysis 2 (two contexts)							
inter-call interval (sqrt)	intercept				1.37	0.07	
	call context (travel hoo)	2	**138**.**03**	**<0**.**000**	−**0**.**71**	**0**.**04**	−**16**.**55**
	sex (male)	1	0.00	0.97	0.00	0.04	−0.03
	age	1	3.34	0.07	−0.00	0.00	−1.85

## 4. Discussion

### 4.1. Acoustic differences relating to the context

Clear acoustic differences were evident for hoos emitted in three different contexts, when callers were either resting, travelling or during alert contexts (seeing a snake). Hoos in two contexts, rest and travel, could be distinguished well from each other, with rest hoos having a longer duration than travel hoos. Around half of hoos emitted in alert contexts had a higher fundamental frequency than the other two contexts. However, the remaining alert hoos showed considerable overlap with hoos from rest and travel contexts. Adding information about the call bout (i.e. inter-call intervals in call sequences), however, increased discrimination considerably, in so far as alert hoos were emitted at lower rates compared to travel hoos, while rest hoos were almost exclusively emitted as single calls. Thus, even though chimpanzee hoos are quiet, inconspicuous calls that sound remarkably similar to each other, we were able to reliably identify different variants, especially when taking call sequences into account.

We found hoo variant discrimination in spite of using a conservative acoustic analysis: we used relatively few acoustic variables (those that could be reliably measured for this quiet call). Also, we used only a subset of individuals to make the discriminant functions, fitting the hoos of remaining individuals according to those discriminant functions. Thus, discriminant functions only took some, not all, individual variation into account. Hence, it is highly likely that our acoustic analysis underestimates the context specificity of these calls. Indeed, in a previous playback experiment, we broadcast *single* rest or alert hoos, eliminating call-interval information. The broadcast hoos nonetheless produced different behavioural responses from chimpanzees depending on the hoo variant broadcast [42,43], suggesting that a single hoo is sufficient for receivers to extract contextual information.

While the distribution of hoos across contexts showed biases between observers and recording devices, the variables observer and recording device were controlled for in the LMMs, so that the demonstrated context effects cannot be accounted for by these variables.

A persistent hypothesis in animal communication is that call variation is a direct reflection of signaller arousal. In this study, we found no clear support for this, as acoustic differences were not consistently related to presumed differences in arousal state. Usually, high compared with low arousal contexts are linked to calls with a high fundamental frequency and rapid rates of emission [54]. Here, the snake context, arguably linked to a relatively high arousal state, elicited hoos with the highest fundamental frequency (alert hoos), but calls were emitted at slower rates than travel hoos (linked with a lower arousal state), suggesting that presumed states of arousal cannot fully explain acoustic characteristics of hoos.

Another key hypothesis in animal communication is that call variation can arise due to ecological adaptation. Calls, for example, that are required to reach receivers at varying distances or across varying habitats with varying patterns of acoustic degradation should be under selection to achieve maximum transmission integrity [55]. Here, however, in all three contexts, signallers and receivers of hoos are at similar (short) distances from each other and in the same habitat. Hence, ecological adaptation of acoustic variation found in these three hoo variants is expected to be minimal [55].

### 4.2. Social motivation to produce hoos

Although the contexts of hoo production differ, callers who emit hoos may share a similar motivation to remain together with receivers in the party. Indeed, previous playback and observational studies examining receiver behaviour in response to hoos emitted in alert, rest and travel contexts suggest that all hoo variants are connected with group cohesion, likely facilitating coordination between signaller and receiver [40,42,43]. Contact calls are common across animal species and are used to coordinate movement in and between animal groups [56]. Both male and female chimpanzees have highly differentiated relationships, showing preferences to associate with kin and non-kin bond partners [34–36]. These preferences probably confer benefits for both males [20] and females [18,29,35] suggesting a selective advantage, and a motivation, to remain associated. In a tropical forest environment, when visibility can be obscured beyond 20 m, vocal cues become vital for maintaining coordination. All three hoo variants are close-range calls unlikely to be audible over 150 m [42,43,57] (C.C. & T.G. 2009, personal observation). Thus, they seem designed to coordinate movement only with individuals close by [40]. Since chimpanzees most often travel with their cooperation partners (kin and bond partners [34,35]), it seems likely that hoos are targeted at these individuals.

Chimpanzees also have long-distance contact calls given while travelling, pant hoots, which promote cohesion. Given that pant hoots can be heard over 500 m [58], they probably promote cohesion of the entire group. Hoos, being quiet calls, can only promote cohesion within subgroups. Chimpanzees are subject to predation from leopards and lethal attacks from neighbouring chimpanzee communities [33,59,60], and thus, selection pressures may also have shaped the use of more ‘private’ calls, resulting in quiet hoos, particularly when individuals are potentially more vulnerable to attack, travelling in small parties.

The chimpanzee fission–fusion social structure creates an additional problem. In each context, whether travel, rest or alert, a different response is required from receivers in order to remain associated. We discuss each of these in turn. When travel hoos are emitted at the start of travelling, they often result in receivers, particularly bond partners of signallers, leaving feeding trees and joining in the travel [40] (C.C. & T.G. 2009, personal observation). Travel hoos sometimes elicit vocal replies, most often as either travel or rest hoos [40] (T.G. 2009, personal observation). With the high possibility of fission in chimpanzees in low visibility habitat, travel hoos seem to promote continued cohesion between specific individuals, especially at moments when fission is most likely, as one individual begins to travel. Thus, if the function of hoos is to maintain cohesion, *receivers* should travel when they hear travel hoos.

Alert hoos function to recruit others to a hidden non-predatory threat, such as snakes or snares [42,43]. Again, they promote cohesion, although alert hoos specifically do so within the vicinity of a threat. Unlike travel hoos, which also promote approach behaviour, alert hoos promote slow, hesitant approach behaviour, an important distinction when approaching a hidden potentially deadly threat.

Rest hoos are emitted principally when stationary and often elicit rest hoos as replies (C.C. & T.G. 2009, personal observation). Rest hoo production typically occurs when individuals are resting out of visibility of each other. Signallers may intermittently emit rest hoos, continuing to rest for some time following rest hoo emission (C.C. & T.G. 2009, personal observation). Sounds of chimpanzee movement can elicit further rest hoos from resting individuals. Since many primate vocalizations are individually distinctive [61], these vocalizations broadcast the continued presence of the signallers and, in addition, probably broadcast the behavioural intention [15,40,62] to remain, although this requires further testing. Thus, if the function of hoos is to maintain cohesion, when receivers hear rest hoos, unlike with travel hoos, they should stay in the vicinity of stationary signallers, and *not* travel.

It seems likely that these three hoo types announce a similar underlying motivational state (to stay together) that can explain why the differing contextual information should be expressed using variants of hoos. However, the question remains, why specifically encode the different contexts within the acoustics of the call type? Why have at least three hoo types?

In alarm call studies, species which have different behavioural escape responses to different predators, such as aerial versus ground predators, often emit acoustically different alarm calls to the different predator classes [5,11,63–65]. This suggests that when selection pressure is high, there may be selection for signallers to produce different signals in order to elicit different behavioural responses from receivers. We extend this logic to non-alarm contexts. If it is adaptive for chimpanzees to coordinate movement with preferred partners, but different contexts require different behaviour from receivers to maintain coordination, low visibility habitat and fission–fusion social structure may promote vocal encoding of context specificity, even in quiet contact-type calls.

Signalling theory states that signals evolve to change receiver behaviour, such that the outcome is favourable for both signaller and receiver [66,67]. To date, this idea has mainly been tested on contexts where signalling is expected to be under strong selection pressure, such as mate attraction [68], offspring begging calls [69,70] and predator contexts [9]. Here, we show a pattern consistent with selection acting on quiet coordination calls to promote different receiver responses in different contexts by encoding contextual differences in the acoustic properties of the calls. This suggests that relatively low-level selection pressures may be sufficient to promote acoustic signals that express specific motivations and elicit specific responses. In the case of hoos, emission is more likely if bond partners are present—in alert [41] and travel [40] contexts, and in travel contexts, bond partners are more likely to join in travel after hearing a travel hoo [40]. Since bond partners are primary cooperation partners [34], hoos may be designed to keep bond partners together to enable cooperation when it is needed. The extent to which coordination driven by benefits gained through cooperative activities explains the evolution of such context-specific call diversification requires further examination.

How much hoo variants are an expression of signallers' behavioural intentions, for example to stay or leave, requires further testing using an intentional framework [3,38,40,41,43,71,72]. If the hoos simply encode the signaller's emotional state, different acoustic properties would be expected, although a sharp distinction between emotion and intention is unwarranted and may not transpire in the acoustic properties of a signal [57]. A question requiring further testing is thus whether or not a degree of intentionality is required to evolve the context specificity described in the acoustic properties of chimpanzee hoos.

## 5. Conclusion

We conclude that even within a single acoustic call type, the hoo, variants of the call are context-specific and can be reliably discriminated using acoustic analysis. Previous playback and observational studies examining receiver behaviour in response to hoos suggest that all three hoo variants are connected with group cohesion, but nonetheless seem to elicit subtly different responses from receivers [40,42,43]. To maintain cohesion, receivers must respond differently to signallers in each context: in rest contexts, receivers must stay in the vicinity of signallers; in travel contexts, receivers must approach signallers; and in alert contexts, receivers must approach signallers with caution. For chimpanzees separated even by short distances in low visibility habitat, visual signals or non-specific vocal signals are likely to be unreliable in maintaining cohesion. One particularly interesting feature of the hoos is the low emotional arousal associated with their production, and that acoustic properties of the three hoo variants cannot be easily explained by emotional state. Relatively low-level selection pressures in the social domain may be sufficient to promote differentiated acoustic signals that encode specific motivations and elicit specific responses. One factor driving the evolution of call diversification may have been the demands of cooperative activities.

## Supplementary Material

Crockford_ESM

## Supplementary Material

Crockford_code

## Supplementary Material

Crockford_simulation

## Supplementary Material

Alert Hoo Series

## Supplementary Material

Rest Hoo Series

## Supplementary Material

Crockford_TravelHoos

## Supplementary Material

Crockford_Data

## Data accessibility

Data and code are available in electronic supplementary material, data file and code file.
