# Supplementary material for: Chimpanzee quiet hoo variants differ according to context
Source: R Soc Open Sci. 2018 May 23;5(5):172066. doi: 10.1098/rsos.172066 (PMC5990785; doi:10.1098/rsos.172066)
Supplement: Crockford_ESM [file rsos172066supp1.docx]

**Chimpanzee quiet hoo variants differ according to context: Supplementary Material**

Catherine Crockford^1,2^*, Thibaud Gruber^,2,3,4^* & Klaus Zuberbühler^2,5,6^

^1^ Max Planck Institute for Evolutionary Anthropology, Department of

Primatology, Leipzig, Germany

^2^ Budongo Conservation Field Station, Masindi, Uganda

^3^ Swiss Center for Affective Sciences, University of Geneva, Switzerland

^4^ University of Oxford, Department of Zoology, Oxford, UK

^5^ University of St Andrews, School of Psychology and Neuroscience, St Andrews, Fife, UK

^6^ Cognitive Science Centre, University of Neuchâtel, Switzerland

*Equal contribution, correspondence should be addressed to:

Catherine Crockford: [crockford@eva.mpg.de](mailto:cc211@st-andrews.ac.uk) and Thibaud Gruber: [thibaud.gruber@gmail.com](mailto:thibaud.gruber@gmail.com)

**Figure S1. The impact of observer and recording device on hoo acoustics.**


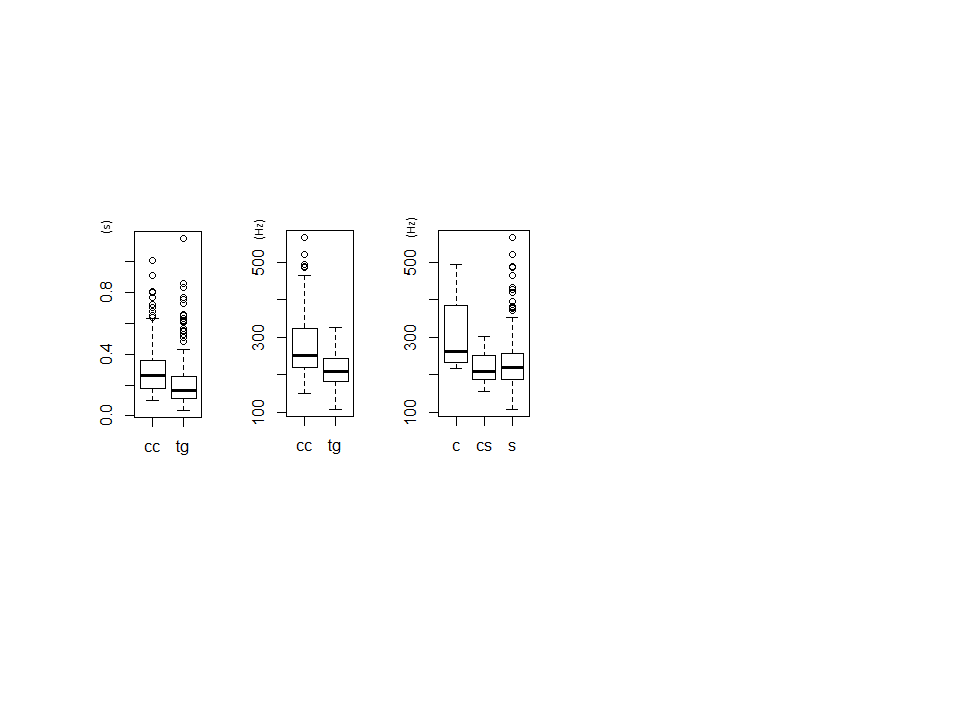


b)

c)

a)

**Legend: a) and b) show the influence of the observer (cc or tg) on call duration (s) and Maximum fundamental frequency (Hz). C) shows the influence of the recording device (c [video camera], cs [video camera + external Sennheiser microphone], s [directional Sennheiser microphone + audio recorder]) on maximum fundamental frequency (Hz).**

**Figure S2. Simulations of model effects, varying the relative effect size of observer and context, using the actual data distribution across observers and contexts.**

**Legend: Results of the simulation with regard to the estimated mean response per context and observer. Effect simulated for (a) observer, (b) context, (c) both. The black horizontal line and boxes show medians and quartiles, and the vertical line depicts the range of the estimated coefficients. The red horizontal line depicts the simulated mean.**

To test whether the LMM we used could reliably disentangle observer and context effects, even when confounded (Table S1), we conducted a simulation. We simulated samples with the same number of calls per observer and context as in our original sample and varied a call feature according to (a) observer, (b) context, or (c) both. In the first simulation (observer effect) we let the simulated call feature be elevated by 1 in one of the observers as compared to the other; in the second simulation (context effect) we simulated a mean response of 0, -0.5, and 0.5 for the three contexts; and in the third simulation we combined simulations one and two. In all three simulations we simulated residual variation by adding numbers randomly drawn from a normal distribution with a mean of zero and a standard deviation of 0.2 to the response. Per simulation we generated 1,000 data sets and fitted a general linear model with the factors observer and context included. We evaluated model results with regard to the estimated mean response per observer and context and the proportion of significant results for the two factors. If the model is able to reliably disentangle observer and context effects the proportions of simulations revealing a type I error [i.e., a significant context effect in model (a) and a significant observer effect in model (b) should be about 0.05 (i.e., the nominal type I error rate) and a high power for the observer effect in models (a) and (c) and the context effect in models (b) and (c)]. Furthermore, we would expect the variation in the estimated coefficients for the same effect (e.g., observer) to be low as compared to the variation between estimated coefficients for different effects.

We found that type I error was close to the nominal 0.05 for the context effect in model (a) (0.046) and also the observer effect in model (b) (0.051). Power for the other effects with simulated effects being different from zero was invariably 1; that is all models revealed a significant effect of the effects simulated to be non-zero. The estimated coefficients varied only little and unbiased (i.e., symmetrically) around the simulated values (**Fig. S2**). Hence, a model in conjunction with data as we had them is able to estimate effects with good precision, has type I error rates at the nominal level, and good power.

**Table S1. Hoo distribution across observers, recording devices and contexts.**

|  | **Alert** | **Rest** | **Travel** |
| --- | --- | --- | --- |
| 1. **Recording device** |  |  |  |
| Video recorder | 13 | 0 | 0 |
| Video recorder + ext mic | 7 | 12 | 0 |
| Mic+ audio-recorder | 20 | 125 | 94 |
|  |  |  |  |
| 1. **Observer** |  |  |  |
| CC | 40 | 46 | 0 |
| TG | 0 | 91 | 94 |
